# Bis{2-(5-hydr­oxy-2-[1-(hydroxy­imino)eth­yl]phenolato-κ^2^ *O* ^1^,*N*}nickel(II) *N*,*N*-dimethyl­formamide disolvate

**DOI:** 10.1107/S1600536809039257

**Published:** 2009-10-03

**Authors:** Yan-Qiu Dang

**Affiliations:** a Department of Chemistry & Chemical Engineering, Binzhou University, Binzhou 256600, People’s Republic of China

## Abstract

The Ni atom of the title complex, [Ni(C_8_H_8_NO_3_)_2_]·2C_3_H_7_NO, lies on a center of inversion in a square-planar N_2_O_2_ coordination environment. An intra­molecular O—H⋯O hydrogen bond exists between the oximic hydr­oxy group of one ligand and the metal-coordinated O atom of the symmetry-related ligand. The dimethyl­formamide solvent mol­ecules are connected to the phenolate groups of the complex *via* O—H⋯O hydrogen bonds.

## Related literature

For general background to the applications of 2-hydroxy­aryl­oxime complexes in extractive metallurgy and biology, see: Keeney *et al.* (1984 ▶); Elo & Lumme (1985 ▶); Chaudhuri (2003 ▶); Milios *et al.* (2007 ▶). For related structures, see: Hatzidimitriou *et al.* (1997 ▶); Voutsas *et al.* (1999 ▶).
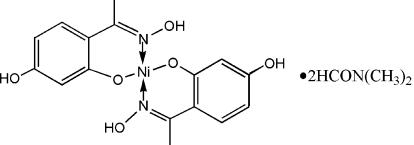


## Experimental

### 

#### Crystal data

[Ni(C_8_H_8_NO_3_)_2_]·2C_3_H_7_NO
*M* *_r_* = 537.21
Monoclinic, 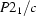

*a* = 13.2905 (10) Å
*b* = 5.8649 (4) Å
*c* = 15.9345 (12) Å
β = 99.129 (1)°
*V* = 1226.32 (16) Å^3^
*Z* = 2
Mo *K*α radiation
μ = 0.85 mm^−1^
*T* = 295 K
0.50 × 0.40 × 0.30 mm

#### Data collection

Bruker SMART APEX area-detector diffractometer
Absorption correction: multi-scan (*SADABS*, Bruker, 2002 ▶) *T*_min_ = 0.677, *T*_max_ = 0.786
6243 measured reflections
2398 independent reflections
2067 reflections with *I* > 2σ(*I*)
*R*_int_ = 0.017

#### Refinement

*R*[*F* ^2^ > 2σ(*F* ^2^)] = 0.028
*wR*(*F* ^2^) = 0.083
*S* = 1.04
2398 reflections
160 parameters
H-atom parameters constrained
Δρ_max_ = 0.25 e Å^−3^
Δρ_min_ = −0.20 e Å^−3^

### 

Data collection: *SMART* (Bruker, 2002 ▶); cell refinement: *SAINT* (Bruker, 2002 ▶); data reduction: *SAINT*; program(s) used to solve structure: *SHELXS97* (Sheldrick, 2008 ▶); program(s) used to refine structure: *SHELXL97* (Sheldrick, 2008 ▶); molecular graphics: *ORTEP-3* (Farrugia, 1997 ▶); software used to prepare material for publication: *SHELXL97*.

## Supplementary Material

Crystal structure: contains datablocks global, I. DOI: 10.1107/S1600536809039257/tk2537sup1.cif

Structure factors: contains datablocks I. DOI: 10.1107/S1600536809039257/tk2537Isup2.hkl

Additional supplementary materials:  crystallographic information; 3D view; checkCIF report

## Figures and Tables

**Table 1:** Hydrogen-bond geometry (Å, °)

*D*—H⋯*A*	*D*—H	H⋯*A*	*D*⋯*A*	*D*—H⋯*A*
O1—H1⋯O4	0.82	1.84	2.622 (2)	159
O3—H3⋯O2^i^	0.82	1.85	2.4857 (19)	134

Symmetry code: (i) 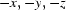.

**Fig. 1.**
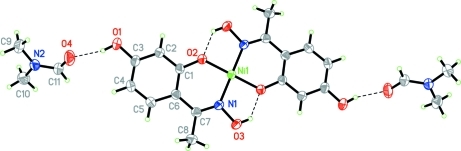
The structure of (I) with displacement ellipsoids are drawn at the 50% probability level. The dashed lines denote hydrogen bonds. The complex is located on a center of inversion; i: -x, -y, -z.

## supplementary crystallographic information

### Comment

2-Hydroxyaryloximes are important organic ligands and their complexes found to have many applications, especially in extractive metallurgy and biology (Chaudhuri, 2003; Elo & Lumme, 1985; Keeney *et al.*, 1984; Milios *et al.*, 2007). Structures of nickel complexes based on the hydroxyoxime ligands 2-[1-(hydroxyimino)ethyl]phenol, bis[2-(1-(hydroxyimino)ethyl)phenolato]nickel (Hatzidimitriou *et al.*, 1997), and bis[2-(5-methyl-1-(hydroxyimino)ethyl)phenolato]nickel (Voutsas *et al.*, 1999), have been reported. As a continuation of these studies, the structure of the title complex, (I), is described herein.

The Ni atom in (I), Fig. 1, is four-coordinate and lies on a center of inversion in a square-planar coordination geometry with the O2—Ni1—N1 angle = 91.84 (6)° and O2—Ni1—N1^i^ = 88.16 (6)°; i: -*x*, -*y*, -*z*. The distances of the Ni1—O2 and Ni1—N1 bonds is similar to those observed in the Ni complexes cited above. The deprotonated phenolato oxygen atom O2 is intramolecularly hydrogen bonded to the oximic hydroxyl group of the opposite ligand, Table 1. The complex and the solvent *N*,*N*-dimethylformamide molecules are linked by the O—H···O hydrogen bonds, Table 1.

### Experimental

Nickel perchlorate hexahydrate (0.36 g, 1 mmol), 1-(2,4-dihydroxyphenyl)ethanone oxime (0.17 g, 1 mmol), H_2_O (6 ml) and DMF (6 ml) were placed in a 20 ml Teflon-lined autoclave. The autoclave was heated at 393 K for 2 days. The autoclave was cooled over a period of 5 h at a rate of 20 K per hour. Green crystals were collected by filtration, washed with methanol, and dried in air; yield 38% based on Ni.

### Refinement

H atoms were placed at calculated positions (C—H = 0.93–0.96 Å and O—H = 0.82 Å) and refined in the riding model approximation with *U*_iso_(H) = 1.2–1.5*U*_eq_(C or O).

### Crystal data

**Table:**

[Ni(C_8_H_8_NO_3_)_2_]·2C_3_H_7_NO	*F*(000) = 564
*M**_r_* = 537.21	*D*_x_ = 1.455 Mg m^−^^3^
Monoclinic, *P*2_1_/*c*	Mo *K*α radiation, λ = 0.71073 Å
Hall symbol: -P 2ybc	Cell parameters from 5624 reflections
*a* = 13.2905 (10) Å	θ = 2.2–26.5°
*b* = 5.8649 (4) Å	µ = 0.85 mm^−^^1^
*c* = 15.9345 (12) Å	*T* = 295 K
β = 99.129 (1)°	Block, brown
*V* = 1226.32 (16) Å^3^	0.50 × 0.40 × 0.30 mm
*Z* = 2	

### Data collection

**Table:**

Bruker SMART APEX area-detector diffractometer	2398 independent reflections
Radiation source: fine-focus sealed tube	2067 reflections with *I* > 2σ(*I*)
graphite	*R*_int_ = 0.017
φ and ω scans	θ_max_ = 26.0°, θ_min_ = 2.6°
Absorption correction: multi-scan (*SADABS*, Bruker, 2002)	*h* = −16→14
*T*_min_ = 0.677, *T*_max_ = 0.786	*k* = −7→6
6243 measured reflections	*l* = −19→13

### Refinement

**Table:**

Refinement on *F*^2^	Primary atom site location: structure-invariant direct methods
Least-squares matrix: full	Secondary atom site location: difference Fourier map
*R*[*F*^2^ > 2σ(*F*^2^)] = 0.028	Hydrogen site location: inferred from neighbouring sites
*wR*(*F*^2^) = 0.083	H-atom parameters constrained
*S* = 1.04	*w* = 1/[σ^2^(*F*_o_^2^) + (0.0436*P*)^2^ + 0.4293*P*] where *P* = (*F*_o_^2^ + 2*F*_c_^2^)/3
2398 reflections	(Δ/σ)_max_ < 0.001
160 parameters	Δρ_max_ = 0.25 e Å^−^^3^
0 restraints	Δρ_min_ = −0.20 e Å^−^^3^

### Special details

**Table:**

Geometry. All e.s.d.'s (except the e.s.d. in the dihedral angle between two l.s. planes) are estimated using the full covariance matrix. The cell e.s.d.'s are taken into account individually in the estimation of e.s.d.'s in distances, angles and torsion angles; correlations between e.s.d.'s in cell parameters are only used when they are defined by crystal symmetry. An approximate (isotropic) treatment of cell e.s.d.'s is used for estimating e.s.d.'s involving l.s. planes.
Refinement. Refinement of *F*^2^ against ALL reflections. The weighted *R*-factor *wR* and goodness of fit *S* are based on *F*^2^, conventional *R*-factors *R* are based on *F*, with *F* set to zero for negative *F*^2^. The threshold expression of *F*^2^ > σ(*F*^2^) is used only for calculating *R*-factors(gt) *etc*. and is not relevant to the choice of reflections for refinement. *R*-factors based on *F*^2^ are statistically about twice as large as those based on *F*, and *R*- factors based on ALL data will be even larger.

### Fractional atomic coordinates and isotropic or equivalent isotropic displacement parameters (Å^2^)

**Table:**

	*x*	*y*	*z*	*U*_iso_*/*U*_eq_	
Ni1	0.0000	0.0000	0.0000	0.03529 (12)	
O1	0.36927 (11)	0.3031 (3)	0.28775 (9)	0.0573 (4)	
H1	0.3831	0.4080	0.3217	0.086*	
O2	0.11298 (11)	0.0218 (2)	0.08068 (10)	0.0512 (4)	
O3	−0.16273 (10)	0.2912 (3)	0.00960 (10)	0.0600 (4)	
H3	−0.1806	0.1977	−0.0281	0.090*	
O4	0.45924 (14)	0.6291 (3)	0.38447 (11)	0.0733 (5)	
N1	−0.06182 (11)	0.2487 (3)	0.04612 (10)	0.0401 (4)	
N2	0.57279 (13)	0.9128 (3)	0.41807 (11)	0.0477 (4)	
C1	0.14012 (14)	0.1839 (3)	0.13806 (11)	0.0370 (4)	
C2	0.23725 (14)	0.1673 (3)	0.18586 (11)	0.0412 (4)	
H2	0.2786	0.0444	0.1773	0.049*	
C3	0.27296 (14)	0.3301 (3)	0.24559 (12)	0.0412 (4)	
C4	0.21144 (16)	0.5124 (3)	0.26013 (14)	0.0472 (5)	
H4	0.2350	0.6225	0.3005	0.057*	
C5	0.11510 (16)	0.5279 (3)	0.21400 (13)	0.0444 (4)	
H5	0.0744	0.6507	0.2241	0.053*	
C6	0.07536 (14)	0.3672 (3)	0.15236 (11)	0.0359 (4)	
C7	−0.02770 (14)	0.3901 (3)	0.10657 (11)	0.0371 (4)	
C8	−0.09685 (17)	0.5748 (4)	0.12913 (15)	0.0539 (5)	
H8A	−0.1664	0.5247	0.1164	0.081*	
H8B	−0.0880	0.7094	0.0968	0.081*	
H8C	−0.0804	0.6085	0.1887	0.081*	
C9	0.62892 (19)	0.7952 (4)	0.49118 (15)	0.0622 (6)	
H9A	0.6381	0.8958	0.5393	0.093*	
H9B	0.6943	0.7492	0.4787	0.093*	
H9C	0.5914	0.6631	0.5038	0.093*	
C10	0.60887 (19)	1.1364 (4)	0.39838 (18)	0.0678 (7)	
H10A	0.6067	1.2368	0.4456	0.102*	
H10B	0.5662	1.1957	0.3490	0.102*	
H10C	0.6777	1.1248	0.3875	0.102*	
C11	0.49409 (16)	0.8175 (4)	0.37059 (14)	0.0533 (5)	
H11	0.4626	0.8977	0.3233	0.064*	

### Atomic displacement parameters (Å^2^)

**Table:**

	*U*^11^	*U*^22^	*U*^33^	*U*^12^	*U*^13^	*U*^23^
Ni1	0.03338 (19)	0.0364 (2)	0.03341 (19)	0.00408 (13)	−0.00300 (12)	−0.00579 (13)
O1	0.0471 (8)	0.0590 (9)	0.0575 (9)	0.0005 (7)	−0.0175 (7)	−0.0137 (7)
O2	0.0461 (8)	0.0486 (8)	0.0520 (8)	0.0128 (6)	−0.0136 (6)	−0.0206 (6)
O3	0.0393 (8)	0.0685 (10)	0.0646 (10)	0.0185 (7)	−0.0148 (7)	−0.0263 (8)
O4	0.0707 (11)	0.0620 (11)	0.0790 (12)	−0.0240 (9)	−0.0133 (9)	−0.0079 (9)
N1	0.0331 (8)	0.0433 (8)	0.0408 (8)	0.0070 (6)	−0.0033 (6)	−0.0046 (7)
N2	0.0420 (9)	0.0450 (9)	0.0548 (10)	−0.0038 (7)	0.0031 (7)	−0.0094 (8)
C1	0.0397 (9)	0.0376 (9)	0.0319 (9)	−0.0009 (8)	0.0000 (7)	−0.0030 (7)
C2	0.0403 (10)	0.0417 (10)	0.0394 (10)	0.0037 (8)	−0.0005 (8)	−0.0039 (8)
C3	0.0404 (10)	0.0440 (10)	0.0364 (9)	−0.0059 (8)	−0.0018 (8)	0.0010 (8)
C4	0.0500 (11)	0.0434 (11)	0.0453 (11)	−0.0072 (9)	−0.0013 (9)	−0.0120 (8)
C5	0.0466 (11)	0.0396 (10)	0.0460 (11)	0.0014 (8)	0.0041 (9)	−0.0084 (8)
C6	0.0391 (9)	0.0363 (9)	0.0319 (9)	−0.0004 (7)	0.0046 (7)	−0.0007 (7)
C7	0.0415 (10)	0.0370 (10)	0.0330 (9)	0.0036 (8)	0.0069 (7)	−0.0004 (7)
C8	0.0506 (12)	0.0528 (12)	0.0563 (13)	0.0135 (10)	0.0025 (10)	−0.0134 (10)
C9	0.0567 (13)	0.0743 (16)	0.0517 (13)	−0.0062 (12)	−0.0034 (10)	−0.0073 (11)
C10	0.0595 (14)	0.0498 (13)	0.0917 (19)	−0.0088 (11)	0.0048 (13)	−0.0066 (12)
C11	0.0470 (11)	0.0536 (12)	0.0553 (12)	−0.0024 (10)	−0.0046 (9)	−0.0093 (10)

### Geometric parameters (Å, °)

**Table:**

Ni1—O2^i^	1.8197 (14)	C3—C4	1.387 (3)
Ni1—O2	1.8197 (14)	C4—C5	1.375 (3)
Ni1—N1^i^	1.8801 (15)	C4—H4	0.9300
Ni1—N1	1.8801 (15)	C5—C6	1.403 (3)
O1—C3	1.357 (2)	C5—H5	0.9300
O1—H1	0.8200	C6—C7	1.452 (2)
O2—C1	1.328 (2)	C7—C8	1.501 (3)
O3—N1	1.3970 (19)	C8—H8A	0.9600
O3—H3	0.8200	C8—H8B	0.9600
O4—C11	1.232 (3)	C8—H8C	0.9600
N1—C7	1.297 (2)	C9—H9A	0.9600
N2—C11	1.315 (3)	C9—H9B	0.9600
N2—C10	1.447 (3)	C9—H9C	0.9600
N2—C9	1.454 (3)	C10—H10A	0.9600
C1—C2	1.394 (2)	C10—H10B	0.9600
C1—C6	1.418 (2)	C10—H10C	0.9600
C2—C3	1.378 (3)	C11—H11	0.9300
C2—H2	0.9300		
			
O2^i^—Ni1—O2	180.00 (14)	C6—C5—H5	118.5
O2^i^—Ni1—N1^i^	91.84 (6)	C5—C6—C1	116.83 (17)
O2—Ni1—N1^i^	88.16 (6)	C5—C6—C7	120.64 (16)
O2^i^—Ni1—N1	88.16 (6)	C1—C6—C7	122.52 (16)
O2—Ni1—N1	91.84 (6)	N1—C7—C6	120.30 (16)
N1^i^—Ni1—N1	180.00 (11)	N1—C7—C8	118.96 (17)
C3—O1—H1	109.5	C6—C7—C8	120.74 (16)
C1—O2—Ni1	129.80 (12)	C7—C8—H8A	109.5
N1—O3—H3	109.5	C7—C8—H8B	109.5
C7—N1—O3	113.25 (14)	H8A—C8—H8B	109.5
C7—N1—Ni1	131.56 (13)	C7—C8—H8C	109.5
O3—N1—Ni1	115.19 (11)	H8A—C8—H8C	109.5
C11—N2—C10	121.4 (2)	H8B—C8—H8C	109.5
C11—N2—C9	121.3 (2)	N2—C9—H9A	109.5
C10—N2—C9	117.25 (18)	N2—C9—H9B	109.5
O2—C1—C2	116.94 (16)	H9A—C9—H9B	109.5
O2—C1—C6	123.20 (16)	N2—C9—H9C	109.5
C2—C1—C6	119.86 (16)	H9A—C9—H9C	109.5
C3—C2—C1	121.11 (17)	H9B—C9—H9C	109.5
C3—C2—H2	119.4	N2—C10—H10A	109.5
C1—C2—H2	119.4	N2—C10—H10B	109.5
O1—C3—C2	117.08 (17)	H10A—C10—H10B	109.5
O1—C3—C4	122.81 (17)	N2—C10—H10C	109.5
C2—C3—C4	120.11 (17)	H10A—C10—H10C	109.5
C5—C4—C3	119.03 (17)	H10B—C10—H10C	109.5
C5—C4—H4	120.5	O4—C11—N2	124.5 (2)
C3—C4—H4	120.5	O4—C11—H11	117.8
C4—C5—C6	123.04 (18)	N2—C11—H11	117.8
C4—C5—H5	118.5		

Symmetry codes: (i) −*x*, −*y*, −*z*.

### Hydrogen-bond geometry (Å, °)

**Table:**

*D*—H···*A*	*D*—H	H···*A*	*D*···*A*	*D*—H···*A*
O1—H1···O4	0.82	1.84	2.622 (2)	159
O3—H3···O2^i^	0.82	1.85	2.4857 (19)	134

Symmetry codes: (i) −*x*, −*y*, −*z*.
